# Patient experiences with electronic medical records: Lessons learned

**DOI:** 10.1002/2327-6924.12170

**Published:** 2014-09-18

**Authors:** Dale Rose, Louiseann T Richter, Jane Kapustin

**Affiliations:** 1 Department of Ambulatory Services, University of Maryland Medical CenterBaltimore, Maryland; 2 School of Advanced Studies, University of PhoenixPhoenix, Arizona; 3 Department of Organizational Systems and Adult Health, University of Maryland School of NursingBaltimore, Maryland

**Keywords:** Communication, nurse practitioners, patient, computers

## Abstract

**Purpose:**

To describe the lived experience of patients communicating with their nurse practitioners and physicians while using paper health records (PHRs) and electronic health records (EHRs) in the examination rooms. The significance of the study lies in the salience of communication between the patient and provider in promoting optimal clinical outcomes and the highest level of patient satisfaction.

**Data sources:**

The study used a qualitative, phenomenological design. Audio-taped focus group interviews were conducted with 21 patients from a diabetes clinic in Baltimore, Maryland. Patients had visits with the provider before and after implementation of EHRs in the clinic.

**Conclusions:**

The four themes that emerged from the three focus groups included communication issues, patient preferences for electronic records, safety and security concerns, and transition problems with implementation of EHRs.

**Implications for practice:**

Potential benefits for nurse practitioners implementing the recommendations in this study include enhanced communication between patients and providers while using EHRs, increased patient satisfaction, higher levels of nurse practitioner and physician satisfaction, and avoidance of communication issues during implementation of EHR systems.

Poor provider–patient communication negatively affects clinical outcomes, including adherence to prescribed medication and treatment regimens, patients’ sense of physical and mental health, patients’ satisfaction with their treatment, patients’ adaption to long-term care, and achievement of a peaceful death (Sheldon & Ellington, 2008). Medical errors caused by healthcare providers can result in unnecessary deaths of thousands of persons each year (Institute of Medicine [IOM], 1999). A report by the IOM in 1999 estimated that 44,000 to 98,000 patients die each year in United States hospitals because of medical errors. Stewart, Kroth, Schuyler, and Bailey (2010) found that implementation of electronic health records (EHRs) affected communication and the relationship between patient and provider.

The conversion from paper to computerized medical records designed to combine data from ancillary services, such as pharmacy, laboratory, and radiology, with various clinical care components, such as nursing plans, administration records, and provider orders, was mandated by the Health Information Technology for Economic and Clinical Health Act (HITECH, 2009). HITECH legislation provides funding for initiatives under the Office of the National Coordinator for Health Information Technology (ONCHIT) and Medicare and Medicaid incentives for hospitals and providers to implement EHRs (Buntin, Jain, & Blumenthal, 2010). Implementation of an EHR system affects many processes associated with patient care, which creates concerns about how use of EHRs affects communication and therapeutic relationships between patients and physicians (Stewart et al., 2010).

Evidence has shown patient–provider communication is the most salient part of a medical visit (Shachak & Reis, 2009). Communication between patient and provider affects patient satisfaction, adherence to prescribed treatment, provider–patient conflict resolution, and clinical outcomes, all of which are important concerns for nurse practitioners. Research studies have connected physician–patient communication to providers’ interpersonal skills in an expanded definition of quality of medical care in patient outcomes.

Shachak and Reis (2009) believed EHRs have a potential for enhancing a shared understanding between doctors and patients on disease management, educating and empowering patients to improve their health status, and improving effectiveness of the medical visit. Because research on the effect of EHRs on provider–patient communication is limited, nurse practitioners may gain salient information from this study.

## Literature review

Frankel et al. (2005) observed the effect of implementation of EHRs in a qualitative, longitudinal study on communication between providers and patients in examination rooms. The study involved videotaping and audio recordings of provider and patient visits before and after implementation of EHRs. Nine clinicians, including primary care physicians, physician assistants, and a nurse practitioner with 54 patients from a multispecialty practice were involved in the study. The study addressed whether use of the EHR improved or interfered with providers’ communication with patients (Frankel et al., 2005).

Sociologists that analyzed the data reported that conversion to EHRs had mixed effects on visual, verbal, and postural relationships between providers and patients (Frankel et al., 2005). Use of EHRs increased complexity of patient flow and organization of a medical visit by adding mental or physical tasks for providers. For providers experienced in using computers and EHRs, software improved the organization of clinical information and streamlined tasks, lessening the complexity of the visit.

Frankel et al. (2005) described examples when the use of EHRs enhanced efficiency of providers with good organizational skills and deteriorated the efficiency of providers with poor organizational skills. In a similar manner, providers with good interpersonal skills were observed to incorporate the use of EHRs without a negative effect on patient–provider communication by the second and third time periods. Clinicians with poor interpersonal skills before using EHRs demonstrated worsening of patient–provider communication with addition of EHRs.

In navigation of EHRs, Frankel et al. (2005) noted that a variety of skills, such as typing and ability to organize information, affected providers with poor skills negatively and clinicians with better skills positively. Arrangement of the computer, monitor, providers’ chair, and examination table varied in rooms used for the study. Configuration of some rooms facilitated alternating attention to patients and EHRs. In other rooms, arrangement of furniture and computers forced clinicians’ to sit facing away from the patients. Frankel et al. (2005) concluded that some of the variation in results among providers could have been from differences in providers’ opinions about EHRs. Some clinicians might have thought of EHRs as tools for rapid documentation of notes and medical orders and other providers could have considered EHRs as an aid in patient education.

Ventres, Kooienga, and Marlin (2006) observed that physicians interacting with patients while using EHRs paid more attention to the computer screen rather than patients. Ventres et al. (2006) noted experienced providers typed on EHRs while patients discussed sensitive concerns, read computer screens with patients waiting in the room, and turned away from patients despite mobile capability of the computer monitors. Physicians used predetermined questions from computer templates rather than using responses from patients (Ventres et al., 2006). Ventres et al. (2006) suggested providers could improve communication with patients by increasing awareness of these behaviors and taking action, such as stopping to listen to patients' concerns, and involving patients by pointing to the screen.

### Satisfaction with provider communication

El-Kareh et al. (2009) conducted a study on EHRs that was implemented in three health centers in Massachusetts. Providers using the EHR were surveyed at four intervals over a year following implementation of the EHR system. Surveys were designed to determine providers' opinions of the effect of EHRs on patient safety, communication between providers, communication with patients, access to care, and efficiency of visits. Eighty-six providers were given surveys, including physicians, nurse practitioners, and physician assistants (El-Kareh et al., 2009). Responses were generally optimistic with a trend of increasingly positive perceptions during the year after implementation (El-Kareh et al., 2009). Perceptions of providers are shown in Table1.

**Table 1:** Clinicians perceptions of EHRs from El-Kareh et al., 2009; N=86

	Percent at	Percent at	
Findings	Start of Year	End of Year	*p* Value
Improved quality of care	63	86	*p* <.001*
Reduced medication errors	72	81	*p* =.03
Enhanced follow-up of test results	62	87	*p* <.001*
Improved communication between providers	72	93	*p* <.001*
Impaired quality of patient–clinician interactions	49	33	*p* =.001*
Lengthened patient visits	68	51	*p* =.001*
Increased time required for documentation	78	68	*p* =.006*

* Findings are statistically significant.

### Quality of care and medical records

According to Sullivan (2010), paper medical records contributed to medical errors made on a daily basis. Sullivan noted medical errors could be attributed to poor handwriting, manual order entry, use of nonstandard abbreviations, and poor legibility. The IOM (1999) report, *Preventing Medication Errors*, estimated every patient was exposed to a potential medical error on every day of a hospital stay. Processes of documentation in electronic medical records can mitigate a potential for medical errors (Sullivan, 2010).

Root causes of medical errors include a lack of shared information among providers for mutual patients according to Sullivan (2010). The lack of communication of information between providers results in repeating tests, ordering unnecessary tests, and delays in care. Implementation of interoperable EHRs could reduce the problem and eliminate associated costs (Sullivan, 2010).

## Methods

This study used a qualitative phenomenological design to explore and understand patient perceptions of provider communication while using paper and electronic medical records. The target population for the study was patients from a diabetes clinic at an urban medical center in Baltimore, Maryland. The size of the population was approximately 1000 patients. Eligibility criteria included adults over the age of 18 who had an office visit with a provider who used a paper health record (PHR) prior to implementation of an EHR system and a subsequent office visit with the same provider who used an EHR. The majority of the population of the clinic was African American. Other groups that comprised the population of the center included Caucasian, Korean, Hispanic, Pacific Islander, and Native American.

Socioeconomic status of the population ranged from homeless to wealthy, unemployed to top executives. A majority of the population lived in the Baltimore metropolitan area. Other patients of the clinic were from Maryland and surrounding states. Tables2 through Table5 provide a demographic analysis of the focus group participants. The geographic location of Baltimore, Maryland, was chosen because the diabetes clinic implemented an EHR in a timeframe in which providers had approximately 2 years to become regular users of the system. Purposive sampling methodology was used because the method elicited participants that had knowledge of the experience of communicating with providers using PHRs and EHRs during visits in an outpatient clinic.

**Table 2:** Demographics: Gender of participants (*N* = 21)

Gender	Number	Percent
Male	8	38
Female	13	62

**Table 3:** Demographics: Age range of participants (*N* = 21)

Age Range	Number of Participants	Percent
35—44	2	10
45—54	4	19
55—64	11	52
65—74	3	14
75—84	1	5

**Table 4:** Demographics: Race/ethnicity of participants (*N* = 21)

Race/Ethnicity	Number of Participants	Percent
African American	12	57
Caucasian/White	8	38
Prefer not to answer	1	5

**Table 5:** Demographics: Education level of participants (*N* = 21)

Education Level	Number of Participants	Percent
Eighth grade	1	5
Some high school	3	14
Completed high school	7	34
Some college	3	14
Associate's degree	3	14
Bachelor's degree	3	14
Master's degree	1	5

### Data collection

During the month prior to the beginning of the study, providers, nurses, and educators in the clinic distributed a letter of invitation to participate in the study to all patients. The letter described the purpose of the study, the method of using focus groups, provided information on the researcher's identity and institutional affiliation, and how to participate in the study. Institutional Review Board (IRB) approval was obtained for the study from the associated universities and medical center prior to data collection. All participants signed an informed consent form prior to participating in the group interview.

Focus group interview questions for this study covered an array of concerns specific to the topic to elicit relevant information. Participants were encouraged to discuss their experiences and associated feelings in-depth. At the beginning of the interviews patients were asked to “Describe how you and your provider communicated during your visit when the provider was using a paper medical record.” Participants were encouraged to share their experiences in a general discussion. Then the discussion was guided toward finding out more details about participants’ experiences. Follow-up questions included (a) What was it like to talk to your doctor or nurse practitioner when they were using paper, and be listened to, what was that experience like? (b) Did you feel like you were heard, and could talk with the doctor or nurse practitioner? (c) How did using the paper affect your expectations of the visit? and (d) Did [the experience] ever affect your satisfaction with the visit? Participants were encouraged to describe their experiences in communicating with nurse practitioners and physicians in responses to the same questions regarding electronic medical records.

Mechanisms were included to make sure participants’ identities or information were not divulged. Pseudonyms were used and inconsequential facts were changed to avoid breaches in confidentiality from occurring, and quotes were kept brief to protect the participants. Audiotaping was used rather than videotaping because the method was less of a threat to exposing participants’ identities. Individual responses to questions in interviews were coded to ensure anonymity and demographic records were kept separately. Participants’ names were not revealed in results of the study. All information pertaining to the study was secured in an office with entry available only to the interviewer. Three focus groups with a size ranging from five (Group 2) to eight (Groups 1 and 3) for a total of 21 participants were conducted to obtain rich data from the patients.

### Data analysis

The analysis involved a process that provided order, structure, and meaning to the data collected from the semistructured interviews. The interviews were electronically recorded and handwritten notes were taken during the interviews. The audio recordings were transcribed and reviewed by the research assistant and researcher to verify accuracy. The most prevalent findings formed the main themes of the lived experiences as perceived by the study population of communicating with providers using PHRs and EHRs. Data saturation was confirmed through checking the consistency of each of the main themes by color coding participant responses in each of the three groups.

## Results

Four main themes of lived experiences in patient–provider communication while providers were using PHRs and EHRs were elicited from study participants. The four themes that emerged from the data analysis were (a) communication issues, (b) patient preferences for EHRs, (c) safety and security concerns, and (d) transition problems. No themes developed among participants of similar socioeconomic or ethnic groups.

### Theme 1: Communication issues

Having eye contact with providers was important to patients in communicating with their physicians and nurse practitioners. Patients perceived eye contact as an indication that providers cared about them. Respondents noticed how much eye contact nurse practitioners and physicians had with patients and participants felt a personal connection was maintained when providers had eye contact while typing on EHRs.

An unexpected subtheme was that patients felt a closer relationship with better communication with nurse practitioners than physicians; patients felt nurse practitioners had more eye contact and listened carefully to patients’ concerns. Patients described how nurse practitioners alternated talking with patients and entering data in EHRs, which made participants feel the nurse practitioners were still paying attention to patients’ needs. As participant 1M, a 55- to 64-year-old African American male who completed high school expressed:

At each visit it was “How are you, what's- any problems, any questions? OK, let's go over this,” and she'd turn to the computer and say “OK, let me keep this all accurate,” and once that was done, turn back to me and talk to me, so I never felt like I was being ignored or not taken care of.

Nurse practitioners explained to patients what they were doing and what to expect when the nurse practitioners were documenting on EHRs. Respondents felt ignored when physicians turned away from patients and typed on EHRs. Patient 2M, a 65- to 74-year-old Caucasian male with a Bachelor's degree said, “you feel like you're being listened to if the doctor or whoever can turn and look at you more than if you see their back.” Participants perceived providers as showing more concern for patients when the physicians or nurse practitioners looked at patients when speaking to them. When providers had their back to patients typing on the computer, patients perceived this position as a barrier to communication.

Patients felt the best positioning of EHRs and individuals was with nurse practitioners or physicians at a small desk with patients sitting next to the desk so that providers could show patients information on the computer and maintain eye contact. In this position, patients said they did not feel ignored. Respondents appreciated when providers had a conversation with patients first about how they were feeling and managing their disease, subsequently reviewed patient medications and documented in EHRs, then talked with patients again. With this routine, patients perceived the potential communication barrier of EHRs was overcome and a personal connection was maintained. Involving patients in documentation and discussion of information such as test results recorded in EHRs made patients feel more involved and comfortable with providers’ use of EHRs during visits.

### Theme 2: Patient preferences for EHRs

In the theme of preferences for EHRs, patients believed the use of EHRs enabled more personal time with their providers, improving the quality of the visit. Patients noticed EHRs reduced the incidence of various providers asking the same questions or nurse practitioners or physicians repeating questions asked in previous visits. Patients liked EHRs because they thought that EHRs did not impair communication with providers. A sense of personal connection and close rapport was maintained in provider–patient relationships while providers typed on the computer. Patients noted that the visits did not seem rushed or longer with the use of EHRs.

Participants liked EHRs because they perceived improvements in care related to enhancements in communication between providers. Physicians and nurse practitioners could easily share information on patients’ medical problems and coordinate patients’ care with other providers via EHRs. Patients noticed that providers had access to test results sooner with EHRs than receiving reports on paper, which participants associated with more responsive care, and participants noticed the benefit of providers using a search function to quickly find relevant test results and view trends of results.

Respondents emphasized how much they appreciated providers’ use of data and trending of test results as a teaching tool. In EHRs, the visual representation of the influence of patients’ self-management behaviors on their diabetes control increased patients’ understanding of their disease process and encouraged patients to comply with their prescribed treatment regimen. As one woman explained:

At first I just thought it was just some silly numbers, but through her showing me on the computer and different things, now I see my A1c is five or as close as possible to five, that I'm doing well … So it helped a lot. It did.

The thickness of PHRs was a symbol for participants as a painful reminder of how sick they were. A respondent stated “Seeing that makes you feel like you're really sick.” Another patient added, “Yeah, I'm like eight inches, you know, and so they've got to pick all that up. So, it's like, I have a hard time just looking at it when they're picking it up, you know?”

Almost all patients agreed with the theme of preference for EHRs. Two respondents stated they liked PHRs better. One participant perceived more eye contact with PHRs. The other patient thought providers took longer to document in EHRs while the providers were getting used to the setup of the screens in EHRs.

### Theme 3: Safety and security concerns

Several safety and security issues were identified by respondents. Lost or misplaced paperwork was a problem in communication with providers using PHRs. Patients were frustrated by providers flipping through a thick file of papers in PHRs, not finding information in PHRs, and the time required for providers to search for information in PHRs. If a test had to be repeated because the report was lost or missing from PHRs, patients were annoyed. Respondents realized this meant wasting time, including a potential delay in detecting a patients’ serious medical problem.

When providers could not find information in PHRs and asked participants if they remembered what was discussed, the issue undermined patients’ confidence in their physician or nurse practitioner. Patients concluded physicians or nurse practitioners not remembering what was previously said and not finding the information in PHRs were an indication the physician or nurse practitioners were bad practitioners. One patient explained:

Well, for me, when I first started here and the doctors talked to me and asked me things, then we would talk about the medications and he would write stuff down, and then when I came back for the next visit, they couldn't find their notes, and they would ask me, well, if I remembered what I discussed, or you know, what the doctor told me and I wasn't trying to remember because I thought the doctor should remember and they were always losing things and you had to do stuff or ask stuff over again, that kind of turned me off. Like, I really, I wanted another doctor. Cause I figured, like, this doctor didn't know what he was doing, losing the paper and all that.

Participants perceived issues with lost or misplaced paperwork as resolved with implementation of EHRs.

Patients recognized that their care was safer when medical records were accurate, such as when the list of medications was precise, up to date, and could be shared with patients’ other providers. Respondents perceived information in EHRs was more accurate in EHRs than PHRs; participants thought EHRs enabled quicker, more precise documentation, and matched up information to the right patients. In PHRs, patients worried about illegibility of handwriting as a barrier to providers reading information correctly.

Participants were aware of safety features of EHRs, such as spell check and having both generic and brand names on medications lists, which increased patients’ confidence that their care would be provided without harm. When providers read back what they typed in EHRs to ask patients for verification of the accuracy of the information, patients felt more secure that their medical records were correct. Respondents noted that providers had easier and faster access to test results and other information previously recorded in EHRs. The data were judged by participants to be more secure in EHRs than PHRs and patients believed information in EHRs was less likely to be misfiled. Patients perceived PHRs as less permanent because of the fragility of paper and ink.

### Theme 4: Transition problems

The current study involved participants who experienced the implementation of EHRs at the clinic in 2008, the year EHRs were first used. Participants were aware nurse practitioners and physicians were learning the EHR system and sensed providers’ frustrations in determining where to document information and how to use specific functions. Patients expressed a feeling of being lost and perceived EHRs as a barrier to communication with providers while they concentrated on learning the system.

Communication between patients and nurse practitioners and physicians was affected by providers’ computer skills in typing without looking at the keyboard. Participants were frustrated with providers’ slow speed in typing, and worried about the accuracy of providers in data entry. Patients expressed a fear that information would be entered incorrectly if they distracted providers. Respondents wondered if the system was implemented too soon and suggested providers needed more training.

## Discussion

Patients perceived many of the nurse practitioners communications techniques as effective in maintaining communication while using EHRs, including establishing rapport with patients by asking how they were feeling and managing their disease, continuing eye contact, and alternating conversation with typing on the computer. When the nurse practitioners explained what they were doing and what to expect, patients’ anxiety was eased. When physicians turned their backs to respondents, patients viewed the action as a barrier to communication. However, participants in this study did not validate Ventres et al.'s (2006) findings that physicians spent more time looking at the computer screen than focusing on patients.

Through interactions with providers during patient visits, participants developed the belief that nurse practitioners and physicians were able to document more quickly on EHRs, providing more personal time for discussion. Patients observed that providers asked the same questions as in a prior visit or repeated questions asked by another nurse practitioner or physician less often, reducing a common frustration in medical visits. Respondents noticed that the electronic data transmission facilitated meeting patients’ goals of obtaining test results more rapidly than providers receiving paper reports and electronic sharing of EHRs among providers promoted communication and collaboration on patients’ treatment plans. The providers’ use of the EHR satisfied patients’ needs and goals and stimulated less negative emotions in participants. Patients’ expressions of their frustrations with PHRs were consistent with the findings of Sullivan (2010) that PHRs contributed to medical errors on a regular basis. Respondents thought their care was safer with EHRs than PHRs.

In findings of the current study, patients vividly expressed the meaning of the implementation of EHRs from their point of view. Participants were acutely aware of the providers’ learning curve in adapting to the system and perceived EHRs as a block to communication until the nurse practitioners and physicians became comfortable with the system and how to use various functions. This left participants feeling very vulnerable during the transition period. Frankel et al. (2005) conducted a longitudinal study involving taping provider and patient visits before and after implementation of EHRs. However, Frankel et al. (2005) eliminated the variable to the effect of the learning curve in adopting EHRs because the providers had previously used the same EHRs in another setting.

When implementing EHRs, nurse practitioners can use the findings of the current study as lessons learned to ease the transition from PHRs to EHRs for nurse practitioners and patients. Specifically, nurse practitioners can be taught the importance of maintaining eye contact with patients. Training nurse practitioners to talk with patients before entering information in EHRs and interspacing documentation and conversation as suggested by patients can assist nurse practitioners and patients in maintaining a personal connection and avoiding the potential for EHRs to create a communication barrier.

Teaching the techniques of involving patients in documentation and discussion of information, such as test results recorded in EHRs, can make patients feel more involved and at ease. Nurse practitioners can emphasize the power of EHRs by explaining how nurse practitioners can use the data and trending in EHRs as a tool to show patients the effects of taking medications, changes in lifestyle, and self-management behaviors can affect patients’ control of the progression of chronic illnesses. Examination rooms can be designed or rearranged to facilitate communication between nurse practitioner and patient.

## Limitations

The qualitative phenomenological study was limited to interviewing 21 patients at the diabetes clinic in Baltimore, Maryland. As with any research using focus groups, the volunteers who participated in the focus group interviews may have changed their responses because of personal opinion, mood, or interest in computers or EHRs. Patients’ responses may have been affected by their physical or emotional state during the interviews and responses of other participants. Participants’ perceptions may have been affected by providers’ learning curve in the implementation of EHRs. Another limitation relates to the current format and sophistication of EHR systems that may evolve in the future, changing patient perceptions of how providers use EHRs and the concurrent effect on provider–patient communication. The limitations of the present study should be considered in conducting future studies of this nature.

## Conclusions

Potential benefits for leaders implementing the recommendations in this study include enhanced communication between patients and providers while using EHRs, increased patient satisfaction, higher levels of provider satisfaction, and avoidance of communication issues during implementation of EHR systems. Training providers in the aforementioned techniques, setting up examination rooms to facilitate providers involving patients in documentation, and using trends in test results as a teaching tool may prevent patient perceptions of EHRs creating barriers to patient–provider communication. Patients may be more satisfied with their medical visits with the improvements in efficiency of documentation, communication between providers, improved accuracy of medication records, and additional education on trends and management of their disease process with the effective use of EHRs. Nurse practitioners may experience less frustration in the transition to EHRs and increased satisfaction with EHRs as a tool to improve time management and quality of patient care.
